# Table of Contents: Fourth International Congress of CiiEM: Health, Well-Being and Ageing in the 21st Century

**DOI:** 10.1080/07853890.2021.1903746

**Published:** 2021-09-28

## Editorial

 vii

## II Key-notes

Aging the death: the importance of having better methods for age at death estimation of old individuals S1

DNA Use in Forensic Human Identification S1

Interactive Technologies in Stroke Recovery: Uncovering Challenges and Opportunities through Physiotherapist's Perspective S2

Might synthetic cannabinoids influence neural differentiation? S2

Modulation of the initial stages of systemic inflammation by intervention in the NFkB signalling pathway S3

Molecular Biomarkers Associated with Respiratory Insufficiency in Amyotrophic Lateral Sclerosis S4

Nursing communication handover in emergency department S5

Pathologic expansion in the C9orf72 gene is associated with accelerated decline of respiratory function and decreased survival in amyotrophic lateral sclerosis S6

Risk indicators in families with abused children and young people: scoping review S6

Role of fibrinogen-erythrocyte and erythrocyte-erythrocyte adhesion on cardiovascular pathologies S9

Satisfaction with nursing care: influence of sociodemographic factors on a sample of hospitalised children S10

Sexual Well-Being in Old Age: Are Older Adults Well Sexually? S11

The oral – systemic link: oral infection/inflammation and the relation to general health S12

## III Free Communications Biochemistry and Biology Biochemistry

Effect of saccharomycin, a natural Saccharomyces cerevisiae biocide, on Hanseniaspora guilliermondii cells surface S12

Interaction between gold nanoparticles and blood proteins S13

Survey of biogenic amines (histamine and spermidine) in commercial seafood by Enzyme Linked Immunosorbent Assay (ELISA) S14

## Biology

Interleukin gene cloning and expression in E. coli S15

The genetic susceptibility linking preterm birth and periodontal disease – A review S16

## Biomedical Sciences

A review on Tumour Treating Fields, a novel modality in cancer treatment S17

Automedication in utentes of municipality of Almada S18

Chemically crosslinked PVA hydrogels for cartilage substitution S19

Discrimination by X-ray fluorescence analysis of elemental concentrations in healthy and diseased rat tissues S19

Hydrogels based on poly(vinyl alcohol) for cartilage substitution S20

Influence of the liquid phase content and presence of hydroxypropyl methyl cellulose on the properties of a calcium phosphate bone cement S21

Layer-by-Layer coated silicone-based soft contact lens hydrogel for diclofenac sustained release S22

Nanoencapsulation as a novel delivery approach for therapeutic applications of gla-rich protein (GRP) S23

Neuromodulation of Lower Limb Motor Pathways with Trans-spinal Direct Current Stimulation: an overview of current findings S24

Physically crosslinked polyvinyl alcohol hydrogels as synthetic cartilage materials S25

Profiles of elemental concentrations in human: contribution of X-ray fluorescence to discrimination between healthy and diseased tissues and prediction of alterations in tongue carcinoma S26

PVA/chitosan hydrogels loaded with octenidine and 2-phenoxyethanol for wound dressings S27

## Chemistry

Direct Esterification of Sucrose: Novel Lead-Compounds Against Cancer S28

Gold(III) bisdithiolate complexes: molecular conductors that also exhibit anticancer and antimicrobial activities S29

Inhibition-based biosensor for cyanide detection – a preliminary study S30

Modification of ZnO nanoparticles with silanes enables their application as anticancer agents S31

## Dental Medicine and Dental Prothesis Dental Medicine

Antibiotic prophylaxis for dental procedures: Do dental students know enough? S32

Assessment of the relationship between oral health-related quality of life (OHRQoL) and dental malocclusion in a Portuguese sample S33

Bacteriophage isolation from human saliva: a pilot study with high school students S34

Bitemarks analysis of orthodontically treated suspects - an identification approach S35

Determination of the Vertical Dimension Occlusion - Case Report S36

Differential Diagnosis of Developmental Defects of Enamel: a review S37

Effect of an antioxidant on the microtensile bond strength (μTBS) of restored teeth after dental bleaching S38

Effect of pH of H2O2 solutions on the morphology and wear resistance of human dental enamel: an AFM study S39

Effect of the saliva biomolecules on the interface zirconia/Ti6Al4V triboactivity S39

Evaluation of an experimental setup to analyse the intermaxillary relation in surfers S41

Evaluation of periodontally diseased molars based on the Miller McEntire Periodontal Prognostic Index S41

Evaluation of Postoperative Pain in Patients Submitted to Periodontal Surgeries S42

Evaluation of Self-perception of Awake Bruxism in Dentistry Students – Clinical Case Series S43

Evaluation of the quality of life before and after rehabilitation with dental implants: a pilot study S44

Evaluation of the relationship between oral health-related quality of life (OHRQoL) and skeletal malocclusion - a pilot study S45

How often are medication prescribed in the emergency appointment at Egas Moniz Dental University Clinic? – A pilot Study S45

*In vitro* erosive dental wear measured with a 3 D intraoral scanner - a pilot study S46

Inflamatory Dentigerous Cyst – A Clinical Case S47

Influence of acid etching on internal bleaching with 16% carbamide peroxide S48

Influence of different antioxidant agents on the microtensile bond strength of restored teeth after bleaching S49

Influence of Silane Type and Application Time on the Bond Strength to Leucite Reinforced Ceramics S50

Low-Level Laser Therapy in neurosensory recovery - Case series approach in dental and maxillofacial rehabilitation S51

Marginal Microleakage of Flowable Resin Composites Used to Adhere Semi-Direct Restorations S52

Multidisciplinary Team in Temporomandibular Disorders S53

My Tooth is Ill: About the Mental Representation of the concept of caries in children (Phase I) S54

Oral Health goes to school S55

Patologia Degenerativa da ATM: Do conservador ao minimamente invasivo - caso clínico S56

Prevalence of imperfect amelogenesis cases in a paediatric population of the paediatric dentistry clinic of IUEM S57

Restorative Materials Without Bis-Gma – Myth or Reality? S58

Sleep Bruxism: the complexity of a definitive diagnosis – Case Report S59

Teeth discolouration and prescribed slimming magistral formula: a case report S60

The child’s self-perception about Dental Decay in the change of Deciduous Teeth S61

The Egas Moniz Histology Digital Platform – a dream that came true S61

The prevalence of patients with rheumatic diseases and its periodontal condition: data from a population-based study in the Lisbon Metropolitan Area S62

Translucency evaluation of an ultra-translucent monolithic zirconia and a super translucent multilayer zirconia: a pilot study on the thickness effect S63

Translucency of HT lithium disilicate specimens with different thicknesses - Preliminary study S64

Treatment of infra-bony periodontal defects using a collagen membrane and a bone substitute of equine origin – a pilot study S65

Vascular Risks in External Sinus Lifts: an Anatomical Approach S66

Vital pulp therapy in a 9-years-old patient: a clinical case S67

What Is the Best Etching Timing in a Resin Infiltrant used on Enamel Surface After Remineralization With CPP-ACP? S68

Temporomandibular disorders in scuba divers: A Systematic Review S69

## Dental Prothesis

3D-Printing of zirconia dental prosthesis S69

Association between mouth-breathing and atypical swallowing in young orthodontic patients at Egas Moniz Dental Clinic S71

## Environmental Sciences

Study of Quantum Dots (CdS, ZnS) toxicity in Danio rerio: preliminary results S72

Study of the effects of nanoplastics ingestion in a freshwater fish (Danio rerio) S73

## Forensic Sciences

2-Phenoxyethanol derivatization in Ink dating determination S74

Assessing the Content of a Package of SGT-151 Sold Online S75

Genetic predisposition for aggressive behaviour related with dopamine and serotonin pathways – An overview S77

Improving methodology to determine Vitamin D3 in food supplements S78

Information System for Tablets Identification (ISTI) S79

Saccharomyces cerevisiae as a model to study synthetic cannabinoids: the impact of using different carbon sources S80

Toxicity of Synthetic Cannabinoids is Increasing Along with the Regulatory Measures Taken for their Control S81

Vehicle identification from automotive paints transferred in road accidents S82

## Health Sciences

Effects of respiratory disease and age in quadriceps muscle mass: a pilot study with ultrasonography S83

Proximity-aware Interactive Displays for Rehabilitation Centres S84

Reliability and validity of the QASCI questionnaire to assess caregiving burden in COPD S85

## Medicine

Anatomical Factors in Medication-Related Osteonecrosis of the Jaws S86

Clinical Outcomes in TMD patients after Arthrocentesis with Lysis, Lavage and Viscossuplementation S87

Collateral circulation in the obstruction of the Superior Vena Cava flow S88

Identification of Potentially Inappropriate Medications with risk of Major Adverse Cardio- and Cerebrovascular Events among elderly patients S89

Infrasound exposure promotes development of atrial fibrosis in rats S90

The Reticular Hypodermic Venous System, the true integrator of the Superficial Venous System  S90

## Microbiology

Comparison of in-office and at-home tooth-whitening products cytotoxicity S91

Effect of Vif in doxorubicin treated breast cancer cells S92

Evaluation of antifungal susceptibility in clinical isolates of the Candida glabrata complex S93

HIV Vif protein in docetaxel treatment of breast cancer cells S94

Microbiological evaluation in oral health units: detection of antibiotic resistant bacteria S95

## Nursing

A Teaching Tool for Nursing Procedure with Oxygen therapy S96

Can prior hospitalisation experiences influence satisfaction with nursing care? Results in a school-aged children sample S97

Motivation factors of nurses in a group of primary health centres in the city of Lisbon: Qualitative Study S98

Nursing Interventions to the Person with Cardiac Disease submitted to ECMO – Integrative Literature Review S99

Quality of Life in Paediatric Oncological Disease: Integrative Literature Review S100

Literature Review on shift work and nursés burnout S100

Strategies used by nurses and tracheostomized users in communication: Systematic Review S101

Work environment and quality of nursing care in primary health care: a scoping review S102

## Nutrition

“Massa de Malagueta”: tradition with a twist S103

Anthropometric and body composition characterisation of competition Portuguese Crossfit athletes S104

Can urban gardens improve food security, health, well-being and financial sustainability of households? S104

Effect of different cooking methods on the content of total vitamin C, ascorbic acid and dehydroascorbic acid of the galega kale S105

Food intake assessment and anthropometric characterisation of teenage gymnasts S106

Influence of temperature and light on total phenolic compounds during natural orange juice storage S107

Milk related excipients in medications: concerns with cow’s milk protein allergy S108

Sexual and reproductive health: The science behind supplementation in aging S109

## Pharmacy

Assessment of the stability of co-amorphous olanzapine in tablets S110

Characterisation and stability of co-amorphous systems containing olanzapine and sulphonic acids S111

Co-formability, solubility enhancement and stability of olanzapine co-amorphous systems produced with different co-formers S112

Comparison of the amorphization ability of two polymorphic forms of olanzapine S113

Cytotoxicity of temperature-responsive cationic diblock copolymers in human cancer and non-cancer cells lines S114

Dosing oral-liquid azithromycin suspensions: do users follow instructions? S115

Evaluation of antibiotic prophylaxis use in a University Dental clinic in the Lisbon Area S116

*In vivo* study on the performance of therapeutic intraocular lens loaded with an antibiotic and an anti-inflammatory S117

Magistral slimming: what are the risks? S118

Polyhexanide and chlorhexidine loaded chitosan wound dressings S119

Resistance Associated Mutations to Protease Inhibitors on HIV-2 infected patients in Portugal S120

Vitamin D in Liquid Food Supplements: are labels in line with RDA? S120

## Psychology: Clinical and Forensic Clinical Psychology

Are the self-esteem and the adult attachment affected by previous experiences of youth victimisation? S121

Do difficulties in emotion regulation impact self-esteem and adult attachment?- The role of trauma S122

Polyvictimization among a juvenile Portuguese sample S123

Predictors of peer victimisation in the context of secondary school S124

The impact of childhood abuse on adult self-esteem and emotional regulation S125

### Forensic Psychology

Elder Abuse: The Hidden Face of Domestic Violence S126

Inmates' empathy: Relationship with childhood victimisation S127

Interpersonal Reactivity: The Impact of Infant-Juvenile Positive Experiences S128

Intimate partner homicide: victims and the dynamics of victim - perpetrator relationship S129

Preliminary Validation of Bumby Rape and Molest Scales: Exploratory Factorial Analysis S130

The reflex of the past – The role of youth victimisation trauma on the interpersonal reactivity S131

Traumatic experiences in a lifetime: Impact on the connection with others and the role of emotions S132

Violence Risk Assessment in Forensic Psychology Office: From Childhood to Elderly S132

Vulnerable Victims in Court: From Childhood to Senescence S133

### Physiotherapy

Adolescent neck pain: association with the use of mobile telephone S134

Ageing and ethical challenges in physiotherapy: Application of the RIPS model in ethical decision-making S135

Analysis of factors related to shoulder instability in young handball players S137

Ankle stiffness assessment in individuals with Chronic Ankle Instability in dual-task single leg stance, on an unstable surface S137

Benefits of a Virtual Environment Program at the level of Functional Physical Fitness in Non-Institutionalized Elderly S138

Benefits of Condylar Distraction in Patients with Temporomandibular Dysfunction S139

Central Synthesis of Temporomandibular Dysfunction S140

Effect of a single dry needling session in Temporomandibular Disorders S141

Effect of auditory neurosensorial stimulation in gait pattern after acute stroke S142

Elderly: Health services and human resources requirements in Portugal S143

Estimating Anatomically Plausible Segment Orientations using a Kinect One Sensor S143

Functional capacity and health status in patient chronic obstructive pulmonary disease S144

Infant massage programs for newborn babies: Systematic Review S145

Modulation of ankle antagonist co-activation during the transition from upright standing to gait and to sit in post-stroke subjects S146

On the Potential of Virtual Reality for Locomotion Rehabilitation S147

Predictors of physical fitness and Health-Related Quality of Life based on anthropometrics characteristics and exercise stress test performance in cardiac rehabilitation S148

Professional Competences of the Physiotherapists in the field of Mental Health in Portugal: a questionnaire based survey S149

Psychosocial aspects in Temporomandibular Disorder: a case report S150

Relationship between handedness and the incidence of spinal changes in the frontal plane: evaluation using Idiag® Spinal Mouse® S151

Relationships between respiratory parameters and quadriceps strength in subjects with chronic obstructive pulmonary disease S152

The Skin temperature under multilayer bandages during exercise S153

Sports Injuries Patterns in Children and Adolescents According to Their Level of Sports Participation, Age and Maturation S154

Sports Practice and Foot Pressure in Children and Teenagers from 10 to 18 Years Old: Evaluation performed using Namrol® Podoprint® S155

Strategies to manage anterior disc displacement without reduction of temporomandibular joint: a case report S156

The effect of progressive aerobic, resistance and stretching exercise combined with education on body weight among breast cancer survivors S157

Translation of Professionalism in Physical Therapy questionnaire for Portuguese of Portugal: Core values S158

### Public Health

Accessing the potential impact of education about cancer screening programs: a pilot before and after study S159

Management of clinical relevant drug-drug interactions with antipsychotics in nursing homes S159

Oral Anticoagulation on patients with atrial fibrillation: are we doing a good job? S160

## Sociology and Social Sciences

Medications in workplace – a literature review S161

