# Correction

**DOI:** 10.1080/07853890.2023.2230422

**Published:** 2023-07-19

**Issue title:** Fourth International Congress of CiiEM: Health, Well-Being and Ageing in the 21st Century

**Journal:** *Annals of Medicine*

**Bibliometrics:** Volume 53, Number S1

**DOI: https://www.tandfonline.com/toc/iann20/53/sup1?nav=tocList**

This supplementary issue published with incorrect page numbers and this has been corrected now as listed below.

II Key-notes

**Table:** 

Aging the death: the importance of having better methods for age at death estimation of old individuals, 10.1080/07853890.2021.1893522	S1
DNA Use in Forensic Human Identification, 10.1080/07853890.2021.1893580,	S2
Interactive Technologies in Stroke Recovery: Uncovering Challenges and Opportunities through Physiotherapist’s Perspective, 10.1080/07853890.2021.1893582,	S3
Might synthetic cannabinoids influence neural differentiation?, 10.1080/07853890.2021.1893997,	S4
Modulation of the initial stages of systemic inflammation by intervention in the NFkB signalling pathway, 10.1080/07853890.2021.1894004	S5
Molecular Biomarkers Associated with Respiratory Insufficiency in Amyotrophic Lateral Sclerosis, 10.1080/07853890.2021.1894009	S6
Nursing communication handover in emergency department, 10.1080/07853890.2021.1896174	S7
Pathologic expansion in the C9orf72 gene is associated with accelerated decline of respiratory function and decreased survival in amyotrophic lateral sclerosis, 10.1080/07853890.2021.1896231	S8
Risk indicators in families with abused children and young people: scoping review, 10.1080/07853890.2021.1896244	S9
Role of fibrinogen-erythrocyte and erythrocyte-erythrocyte adhesion on cardiovascular pathologies, 10.1080/07853890.2021.1896875	S12
Satisfaction with nursing care: influence of sociodemographic factors on a sample of hospitalised children, 10.1080/07853890.2021.1896876	S13
Sexual Well-Being in Old Age: Are Older Adults Well Sexually?, 10.1080/07853890.2021.1896878	S14
The oral – systemic link: oral infection/inflammation and the relation to general health, 10.1080/07853890.2021.1896879	S15

III Free Communications

Biochemistry and Biology

Biochemistry

**Table:** 

Effect of saccharomycin, a natural Saccharomyces cerevisiae biocide, on Hanseniaspora guilliermondii cells surface, 10.1080/07853890.2021.1896881	S16
Interaction between gold nanoparticles and blood proteins, 10.1080/07853890.2021.1896884	S18
Survey of biogenic amines (histamine and spermidine) in commercial seafood by Enzyme Linked Immunosorbent Assay (ELISA), 10.1080/07853890.2021.1896888	S19

Biology

**Table:** 

Interleukin gene cloning and expression in E. coli, 10.1080/07853890.2021.1896889	S20
The genetic susceptibility linking preterm birth and periodontal disease – A review, 10.1080/07853890.2021.1896890	S21

Biomedical Sciences

**Table:** 

A review on Tumour Treating Fields, a novel modality in cancer treatment, 10.1080/07853890.2021.1896891	S23
Automedication in utentes of municipality of Almada, 10.1080/07853890.2021.1896892	S24
Chemically crosslinked PVA hydrogels for cartilage substitution, 10.1080/07853890.2021.1896893	S25
Discrimination by X-ray fluorescence analysis of elemental concentrations in healthy and diseased rat tissues, 10.1080/07853890.2021.1896894	S26
Hydrogels based on poly(vinyl alcohol) for cartilage substitution, 10.1080/07853890.2021.1896896	S27
Influence of the liquid phase content and presence of hydroxypropyl methyl cellulose on the properties of a calcium phosphate bone cement, 10.1080/07853890.2021.1896897	S28
Layer-by-Layer coated silicone-based soft contact lens hydrogel for diclofenac sustained release, 10.1080/07853890.2021.1896898	S30
Nanoencapsulation as a novel delivery approach for therapeutic applications of gla-rich protein (GRP), 10.1080/07853890.2021.1896900	S31
Neuromodulation of Lower Limb Motor Pathways with Trans-spinal Direct Current Stimulation:an overview of current findings, 10.1080/07853890.2021.1896902	S32
Physically crosslinked polyvinyl alcohol hydrogels as synthetic cartilage materials, 10.1080/07853890.2021.1896904	S33
Profiles of elemental concentrations in human: contribution of X-ray fluorescence to discrimination between healthy and diseased tissues and prediction of alterations in tongue carcinoma, 10.1080/07853890.2021.1896905	S34
PVA/chitosan hydrogels loaded with octenidine and 2-phenoxyethanol for wound dressings, 10.1080/07853890.2021.1896910	S35

Chemistry

**Table:** 

Direct Esterification of Sucrose: Novel Lead-Compounds Against Cancer, 10.1080/07853890.2021.1896911	S37
Gold(III) bisdithiolate complexes: molecular conductors that also exhibit anticancer and antimicrobial activities, 10.1080/07853890.2021.1896913	S39
Inhibition-based biosensor for cyanide detection – a preliminary study, 10.1080/07853890.2021.1896915	S41
Modification of ZnO nanoparticles with silanes enables their application as anticancer agents, 10.1080/07853890.2021.1896916	S43

Dental Medicine and Dental Prothesis

Dental Medicine

**Table:** 

Antibiotic prophylaxis for dental procedures: Do dental students know enough?, 10.1080/07853890.2021.1897460	S44
Assessment of the relationship between oral health-related quality of life (OHRQoL) and dental malocclusion in a Portuguese sample, 10.1080/07853890.2021.1896918	S45
Bacteriophage isolation from human saliva: a pilot study with high school students, 10.1080/07853890.2021.1896919	S46
Bitemarks analysis of orthodontically treated suspects - an identification approach, 10.1080/07853890.2021.1896921	S47
Determination of the Vertical Dimension Occlusion - Case Report, 10.1080/07853890.2021.1896930	S48
Differential Diagnosis of Developmental Defects of Enamel: a review, 10.1080/07853890.2021.1897305	S49
Effect of an antioxidant on the microtensile bond strength (lTBS) of restored teeth after dental bleaching, 10.1080/07853890.2021.1897310	S51
Effect of pH of H2O2 solutions on the morphology and wear resistance of human dental enamel: an AFM study, 10.1080/07853890.2021.1897311	S52
Effect of the saliva biomolecules on the interface zirconia/Ti6Al4V triboactivity, 10.1080/07853890.2021.1897312	S53
Evaluation of an experimental setup to analyse the intermaxillary relation in surfers, 10.1080/07853890.2021.1897318	S55
Evaluation of periodontally diseased molars based on the Miller McEntire Periodontal Prognostic Index, 10.1080/07853890.2021.1897342	S56
Evaluation of Postoperative Pain in Patients Submitted to Periodontal Surgeries, 10.1080/07853890.2021.1897343	S57
Evaluation of Self-perception of Awake Bruxism in Dentistry Students – Clinical Case Series, 10.1080/07853890.2021.1897344	S58
Evaluation of the quality of life before and after rehabilitation with dental implants: a pilot study, 10.1080/07853890.2021.1897345	S59
Evaluation of the relationship between oral health-related quality of life (OHRQoL) and skeletal malocclusion - a pilot study, 10.1080/07853890.2021.1897346	S60
How often are medication prescribed in the emergency appointment at Egas Moniz Dental University Clinic? – A pilot Study, 10.1080/07853890.2021.1897347	S61
In vitro erosive dental wear measured with a 3 D intraoral scanner – a pilot study, 10.1080/07853890.2021.1897348	S62
Inflamatory Dentigerous Cyst – A Clinical Case, 10.1080/07853890.2021.1897349	S64
Influence of acid etching on internal bleaching with 16% carbamide peroxide, 10.1080/07853890.2021.1897351	S65
Influence of different antioxidant agents on the microtensile bond strength of restored teeth after bleaching, 10.1080/07853890.2021.1897355	S66
Influence of Silane Type and Application Time on the Bond Strength to Leucite Reinforced Ceramics, 10.1080/07853890.2021.1897360	S67
Low-Level Laser Therapy in neurosensory recovery - Case series approach in dental and maxillofacial rehabilitation, 10.1080/07853890.2021.1897361	S68
Marginal Microleakage of Flowable Resin Composites Used to Adhere Semi-Direct Restorations, 10.1080/07853890.2021.1897362	S69
Multidisciplinary Team in Temporomandibular Disorders, 10.1080/07853890.2021.1897363	S70
My Tooth is Ill: About the Mental Representation of the concept of caries in children (Phase I), 10.1080/07853890.2021.1897365	S71
Oral Health goes to school, 10.1080/07853890.2021.1897366	S72
Degenerative joint disease: From a conservative to a minimally invasive approach – clinical case, 10.1080/07853890.2021.1897368	S74
Prevalence of imperfect amelogenesis cases in a paediatric population of the paediatric dentistry clinic of IUEM, 10.1080/07853890.2021.1897376	S75
Restorative Materials Without Bis-Gma – Myth or Reality?, 10.1080/07853890.2021.1897387	S76
Sleep Bruxism: the complexity of a definitive diagnosis – Case Report, 10.1080/07853890.2021.1897389	S77
Teeth discolouration and prescribed slimming magistral formula: a case report, 10.1080/07853890.2021.1897390	S78
The child’s self-perception about Dental Decay in the change of Deciduous Teeth, 10.1080/07853890.2021.1897393	S79
The Egas Moniz Histology Digital Platform – a dream that came true, 10.1080/07853890.2021.1897395	S80
The prevalence of patients with rheumatic diseases and its periodontal condition: data from a population-based study in the Lisbon Metropolitan Area, 10.1080/07853890.2021.1897396	S81
Translucency evaluation of an ultra-translucent monolithic zirconia and a super translucent multilayer zirconia: a pilot study on the thickness effect¸ 10.1080/07853890.2021.1897400	S82
Translucency of HT lithium disilicate specimens with different thicknesses - Preliminary Study, 10.1080/07853890.2021.1897401	S83
Treatment of infra-bony periodontal defects using a collagen membrane and a bone substitute of equine origin – a pilot study, 10.1080/07853890.2021.1897404	S84
Vascular Risks in External Sinus Lifts: an Anatomical Approach, 10.1080/07853890.2021.1897405	S85
Vital pulp therapy in a 9-years-old patient: a clinical case, 10.1080/07853890.2021.1897414	S86
What Is the Best Etching Timing in a Resin Infiltrant used on Enamel Surface After Remineralization With CPP-ACP?, 10.1080/07853890.2021.1897416	S87
Temporomandibular disorders in scuba divers: A Systematic Review, 10.1080/07853890.2021.1897419	S88

Dental Prothesis

**Table:** 

3D-Printing of zirconia dental prosthesis, 10.1080/07853890.2021.1897420	S89
Association between mouth-breathing and atypical swallowing in young orthodontic patients at Egas Moniz Dental Clinic, 10.1080/07853890.2021.1897421	S91

Environmental Sciences

**Table:** 

Study of Quantum Dots (CdS, ZnS) toxicity in Danio rerio: preliminary results, 10.1080/07853890.2021.1897422	S92
Study of the effects of nanoplastics ingestion in a freshwater fish (Danio rerio), 10.1080/07853890.2021.1897423	S93

Forensic Sciences

**Table:** 

2-Phenoxyethanol derivatization in Ink dating determination, 10.1080/07853890.2021.1897424	S95
Assessing the Content of a Package of SGT-151 Sold Online, 10.1080/07853890.2021.1897425	S97
Genetic predisposition for aggressive behaviour related with dopamine and serotonin pathways – An overview, 10.1080/07853890.2021.1897426	S99
Improving methodology to determine Vitamin D3 in food supplements, 10.1080/07853890.2021.1897427	S100
Information System for Tablets Identification (ISTI), 10.1080/07853890.2021.1897428	S102
Saccharomyces cerevisiae as a model to study synthetic cannabinoids: the impact of using different carbon sources, 10.1080/07853890.2021.1897429	S103
Toxicity of Synthetic Cannabinoids is Increasing Along with the Regulatory Measures Taken for their Control, 10.1080/07853890.2021.1897430	S105
Vehicle identification from automotive paints transferred in road accidents, 10.1080/07853890.2021.1897431	S107

Health Sciences

**Table:** 

Effects of respiratory disease and age in quadriceps muscle mass: a pilot study with ultrasonography, 10.1080/07853890.2021.1897436	S108
Proximity-aware Interactive Displays for Rehabilitation Centres, 10.1080/07853890.2021.1897439	S110
Reliability and validity of the QASCI questionnaire to assess caregiving burden in COPD, 10.1080/07853890.2021.1897441	S111

Medicine

**Table:** 

Anatomical Factors in Medication-Related Osteonecrosis of the Jaws, 10.1080/07853890.2021.1897445	S113
Clinical Outcomes in TMD patients after Arthrocentesis with Lysis, Lavage and Viscossuplementation, 10.1080/07853890.2021.1897446	S114
Collateral circulation in the obstruction of the Superior Vena Cava flow, 10.1080/07853890.2021.1897447	S115
Identification of Potentially Inappropriate Medications with risk of Major Adverse Cardioand Cerebrovascular Events among elderly patients, 10.1080/07853890.2021.1897448	S116
Infrasound exposure promotes development of atrial fibrosis in rats, 10.1080/07853890.2021.1897455	S117
The Reticular Hypodermic Venous System, the true integrator of the Superficial Venous System, 10.1080/07853890.2021.1897456	S118

Microbiology

**Table:** 

Comparison of in-office and at-home tooth-whitening products cytotoxicity, 10.1080/07853890.2021.1895580	S119
Effect of Vif in doxorubicin treated breast cancer cells, 10.1080/07853890.2021.1895582	S120
Evaluation of antifungal susceptibility in clinical isolates of the Candida glabrata complex, 10.1080/07853890.2021.1895583	S122
HIV Vif protein in docetaxel treatment of breast cancer cells, 10.1080/07853890.2021.1895585	S123
Microbiological evaluation in oral health units: detection of antibiotic resistant bacteria, 10.1080/07853890.2021.1895586	S124

Nursing

**Table:** 

A Teaching Tool for Nursing Procedure with Oxygen therapy, 10.1080/07853890.2021.1895964	S125
Can prior hospitalisation experiences influence satisfaction with nursing care? Results in a school-aged children sample, 10.1080/07853890.2021.1895966	S126
Motivation factors of nurses in a group of primary health centres in the city of Lisbon: Qualitative Study, 10.1080/07853890.2021.1895984	S127
Nursing Interventions to the Person with Cardiac Disease submitted to ECMO – Integrative Literature Review, 10.1080/07853890.2021.1896014	S128
Quality of Life in Paediatric Oncological Disease: Integrative Literature Review, 10.1080/07853890.2021.1896045	S129
Literature Review on shift work and nurse’s burnout, 10.1080/07853890.2021.1897458	S130
Strategies used by nurses and tracheostomized users in communication: Systematic Review, 10.1080/07853890.2021.1896071	S131
Work environment and quality of nursing care in primary health care: a scoping review, 10.1080/07853890.2021.1896072	S132

Nutrition

**Table:** 

“Massa de Malagueta”: tradition with a twist, 10.1080/07853890.2021.1896073		S133
Anthropometric and body composition characterisation of competition Portuguese Crossfit athletes, 10.1080/07853890.2021.1896075		S134
Can urban gardens improve food security, health, well-being and financial sustainability of households?, 10.1080/07853890.2021.1896077		S135
Effect of different cooking methods on the content of total vitamin C, ascorbic acid and dehydroascorbic acid of the galega kale, 10.1080/07853890.2021.1896080		S136
Food intake assessment and anthropometric characterisation of teenage gymnasts, 10.1080/07853890.2021.1896081		S137
Influence of temperature and light on total phenolic compounds during natural orange juice storage, 10.1080/07853890.2021.1896082		S138
Milk related excipients in medications: concerns with cow’s milk protein allergy, 10.1080/07853890.2021.1896091		S139
Sexual and reproductive health: The science behind supplementation in aging, 10.1080/07853890.2021.1896092		S140

Pharmacy

**Table:** 

Assessment of the stability of co-amorphous olanzapine in tablets, 10.1080/07853890.2021.1896097	S141
Characterisation and stability of co-amorphous systems containing olanzapine and sulphonic acids, 10.1080/07853890.2021.1896098	S143
Co-formability, solubility enhancement and stability of olanzapine co-amorphous systems produced with different co-formers, 10.1080/07853890.2021.1896099	S144
Comparison of the amorphization ability of two polymorphic forms of olanzapine, 10.1080/07853890.2021.1896100	S146
Cytotoxicity of temperature-responsive cationic diblock copolymers in human cancer and non-cancer cells lines, 10.1080/07853890.2021.1896101	S148
Dosing oral-liquid azithromycin suspensions: do users follow instructions?, 10.1080/07853890.2021.1896105	S150
Evaluation of antibiotic prophylaxis use in a University Dental clinic in the Lisbon Area, 10.1080/07853890.2021.1897459	S151
In vivo study on the performance of therapeutic intraocular lens loaded with an antibiotic and an anti-inflammatory, 10.1080/07853890.2021.1896109	S152
Magistral slimming: what are the risks?, 10.1080/07853890.2021.1896111	S153
Polyhexanide and chlorhexidine loaded chitosan wound dressings, 10.1080/07853890.2021.1896112	S155
Resistance Associated Mutations to Protease Inhibitors on HIV-2 infected patients in Portugal, 10.1080/07853890.2021.1896142	S156
Vitamin D in Liquid Food Supplements: are labels in line with RDA?, 10.1080/07853890.2021.1896145	S157

Psychology: Clinical and Forensic

Clinical Psychology

**Table:** 

Are the self-esteem and the adult attachment affected by previous experiences of youth victimisation?, 10.1080/07853890.2021.1896148	S158
Do difficulties in emotion regulation impact self-esteem and adult attachment?-The role of trauma, 10.1080/07853890.2021.1896150	S159
Polyvictimization among a juvenile Portuguese sample, 10.1080/07853890.2021.1896152	S161
Predictors of peer victimisation in the context of secondary school, 10.1080/07853890.2021.1896153	S162
The impact of childhood abuse on adult self-esteem and emotional regulation, 10.1080/07853890.2021.1896171	S164

Forensic Psychology

**Table:** 

Elder Abuse: The Hidden Face of Domestic Violence, 10.1080/07853890.2021.1896175	S165
Inmates’ empathy: Relationship with childhood victimisation, 10.1080/07853890.2021.1896176	S166
Interpersonal Reactivity: The Impact of Infant-Juvenile Positive Experiences, 10.1080/07853890.2021.1896177	S167
Intimate partner homicide: victims and the dynamics of victim – perpetrator Relationship, 10.1080/07853890.2021.1896181	S168
Preliminary Validation of Bumby Rape and Molest Scales: Exploratory Factorial Analysis, 10.1080/07853890.2021.1896190	S169
The reflex of the past – The role of youth victimisation trauma on the interpersonal Reactivity, 10.1080/07853890.2021.1896192	S170
Traumatic experiences in a lifetime: Impact on the connection with others and the role of emotions, 10.1080/07853890.2021.1896197	S171
Violence Risk Assessment in Forensic Psychology Office: From Childhood to Elderly, 10.1080/07853890.2021.1896198	S172
Vulnerable Victims in Court: From Childhood to Senescence, 10.1080/07853890.2021.1896199	S173

Physiotherapy

**Table:** 

Adolescent neck pain: association with the use of mobile telephone, 10.1080/07853890.2021.1896436	S174
Ageing and ethical challenges in physiotherapy: Application of the RIPS model in ethical decision-making, 10.1080/07853890.2021.1896437	S175
Analysis of factors related to shoulder instability in young handball players, 10.1080/07853890.2021.1896439	S177
Ankle stiffness assessment in individuals with Chronic Ankle Instability in dual-task single leg stance, on an unstable surface, 10.1080/07853890.2021.1896440	S178
Benefits of a Virtual Environment Program at the level of Functional Physical Fitness in Non-Institutionalized Elderly, 10.1080/07853890.2021.1896441	S179
Benefits of Condylar Distraction in Patients with Temporomandibular Dysfunction, 10.1080/07853890.2021.1896442	S180
Central Synthesis of Temporomandibular Dysfunction, 10.1080/07853890.2021.1896443	S181
Effect of a single dry needling session in Temporomandibular Disorders, 10.1080/07853890.2021.1896460	S182
Effect of auditory neurosensorial stimulation in gait pattern after acute stroke, 10.1080/07853890.2021.1896544	S183
Elderly: Health services and human resources requirements in Portugal, 10.1080/07853890.2021.1896551	S184
Estimating Anatomically Plausible Segment Orientations using a Kinect One Sensor, 10.1080/07853890.2021.1896569	S185
Functional capacity and health status in patient chronic obstructive pulmonary disease, 10.1080/07853890.2021.1896586	S187
Infant massage programs for newborn babies: Systematic Review, 10.1080/07853890.2021.1896600	S188
Modulation of ankle antagonist co-activation during the transition from upright standing to gait and to sit in post-stroke subjects, 10.1080/07853890.2021.1896620	S189
On the Potential of Virtual Reality for Locomotion Rehabilitation, 10.1080/07853890.2021.1896637	S191
Predictors of physical fitness and Health-Related Quality of Life based on Anthropometrics characteristics and exercise stress test performance in cardiac rehabilitation, 10.1080/07853890.2021.1896644	S192
Professional Competences of the Physiotherapists in the field of Mental Health in Portugal:a questionnaire based survey, 10.1080/07853890.2021.1896645	S193
Psychosocial aspects in Temporomandibular Disorder: a case report, 10.1080/07853890.2021.1896646	S194
Relationship between handedness and the incidence of spinal changes in the frontal plane: evaluation using Idiag® Spinal Mouse®, 10.1080/07853890.2021.1896654	S195
Relationships between respiratory parameters and quadriceps strength in subjects with chronic obstructive pulmonary disease, 10.1080/07853890.2021.1896688	S196
The Skin temperature under multilayer bandages during exercise, 10.1080/07853890.2021.1896730	S197
Sports Injuries Patterns in Children and Adolescents According to Their Level of Sports Participation, Age and Maturation, 10.1080/07853890.2021.1896775	S199
Sports Practice and Foot Pressure in Children and Teenagers from 10 to 18 Years Old: Evaluation performed using Namrol® Podoprint®, 10.1080/07853890.2021.1896796	S200
Strategies to manage anterior disc displacement without reduction of temporomandibular joint: a case report, 10.1080/07853890.2021.1896838	S201
The effect of progressive aerobic, resistance and stretching exercise combined with education on body weight among breast cancer survivors, 10.1080/07853890.2021.1896839	S202
Translation of Professionalism in Physical Therapy questionnaire for Portuguese of Portugal: Core values, 10.1080/07853890.2021.1896840	S203

Public Health

**Table:** 

Accessing the potential impact of education about cancer screening programs: a pilot, before and after study, 10.1080/07853890.2021.1896842	S204
Management of clinical relevant drug-drug interactions with antipsychotics in nursing homes, 10.1080/07853890.2021.1896843	S205
Oral Anticoagulation on patients with atrial fibrillation: are we doing a good job?, 10.1080/07853890.2021.1896844	S206

Sociology and Social Sciences

**Table:** 

Medications in workplace – a literature review, 10.1080/07853890.2021.1896846	S207

